# Spatially and temporally continuous estimates of annual total nitrogen deposition over North America, 1860–2013

**DOI:** 10.1016/j.dib.2017.12.052

**Published:** 2018-01-03

**Authors:** Robbie A. Hember

**Affiliations:** Climate Change and Integrated Planning Branch, BC Ministry of Forests, Lands, Natural Resource Operations and Rural Development, Canada

## Abstract

This report describes the North American Climate Integration and Diagnostics – Nitrogen Deposition Version 1 (NACID-NDEP1) database. The database contains estimates of annual total nitrogen (N) deposition for the purpose of supporting terrestrial ecosystem modelling in North America. It was constructed at 1-km resolution with coverage of Alaska, Canada, and the conterminous U.S., with continuous annual coverage from 1860 to 2013. Estimates were produced by acquiring and compiling best-available data sources: Wet N deposition was estimated from interpolation of monthly ammonium and nitrate concentration measurements and from grids of monthly precipitation. Dry N deposition was estimated from satellite measurements of ammonium and nitrogen oxides. Total N deposition for the pre-industrial era was derived from previous modelling studies. As these source datasets covered different time periods, several assumptions were required to produce a continuous record.

**Specifications Table**

**Table:** 

Subject area	*Physical geography*
More specific subject area	*Atmosphere-surface nitrogen cycle*
Type of data	*Raster (Geotiff)*
How data was acquired	*Station measurements and remote sensing*
Data format	*Analyzed*
Experimental factors	*A combination of different observations and simulations were compiled to produce continuous maps of annual total nitrogen deposition*
Experimental features	*Moderate-resolution maps of annual total nitrogen deposition and continuous time series of annual total nitrogen deposition*
Data source location	*Alaska, Canada, conterminous U.S.*
Data accessibility	*Mendeley – NACID-NDEP1*
Related research article	*Hember R.A., Kurz, W.A., Coops, N.C., in press. Statistical performance and behaviour of environmentally-sensitive composite models of lodgepole pine growth. Forest Ecology and Management.*

**Value of the Data**

• Demand for continuous, high-resolution environmental data is growing. The necessary data commonly exist, but they often need to be compiled from different agencies and combined to produce a single database.
• Estimates include coverage of relatively remote regions not presently available in most other available products.
• They can be used to estimate the baseline input of nitrogen in nitrogen addition experiments, the development of critical load maps, and to drive models of plant productivity.
• The dataset is spatially synchronized to a larger database of environmental variables, as part of the NACID project.

## 1 Data

### 1.1 Wet deposition

**Fig. 1:**
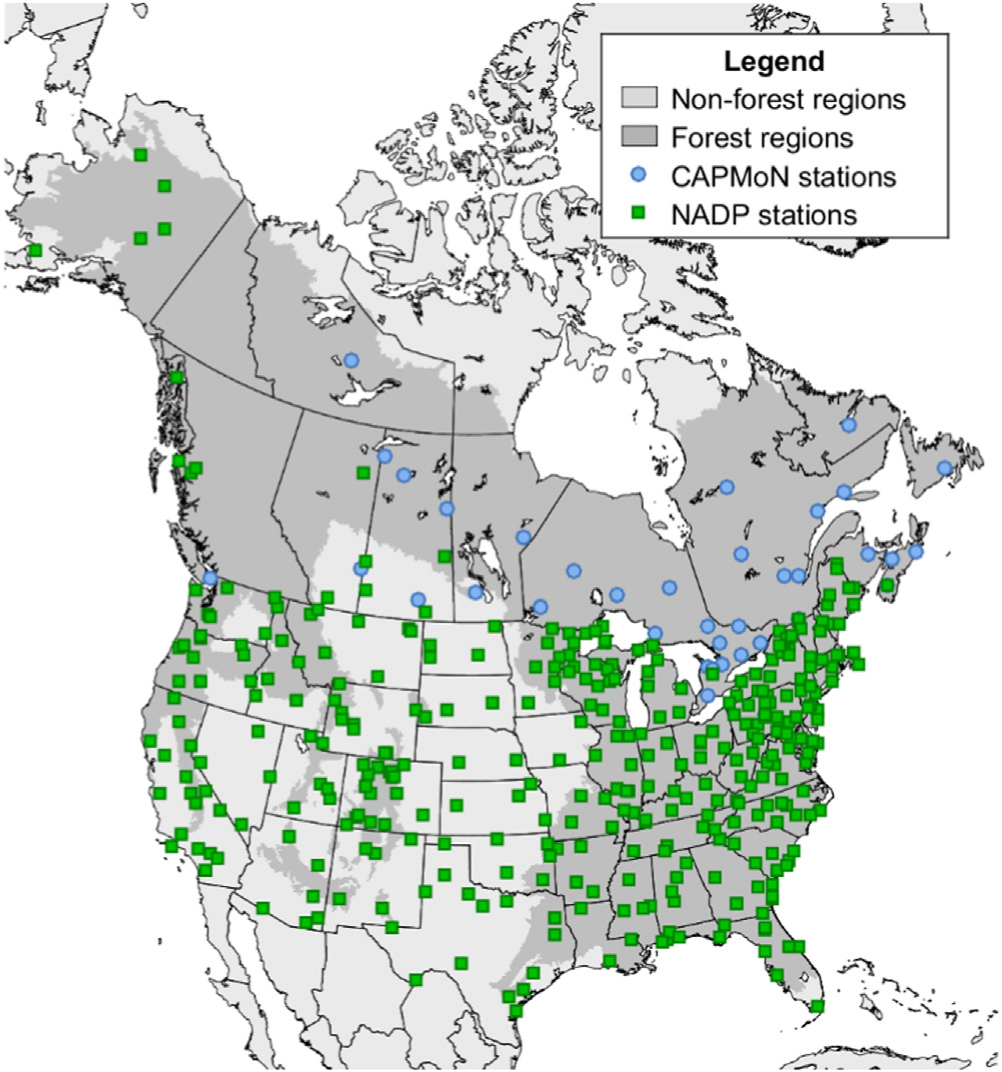
Station measurements of wet nitrogen deposition.

Discontinuous measurements of wet N deposition were conducted by agencies in Canada and the U.S. between 1990 and 2013. In Canada, wet N deposition measurements were collected across the Canadian Air and Precipitation Monitoring Network, consisting of 33 stations distributed across Canada (Fig. 1). The network collected hourly concentrations of $N{O_{3}}^{-}$ and $N{H_{4}}^{+}$ in precipitation. Data were requested and received from Environment Canada in 2015 [1]. The data consisted of monthly (precipitation-weighted) mean concentration summaries (mg l^−1^) by station and time-step. The timespan of records varied by station. The earliest start year was 1990 and the latest available end year was 2013. Months with missing hourly measurements, constituting approximately 14 percent of the monthly data, were not gap-filled. In the U.S., measurements of the concentration of $N{O_{3}}^{-}$ and $N{H_{4}}^{+}$ in precipitation were downloaded from the National Trends Network summary of the National Atmospheric Deposition Program database [2]. Stations in NTN summary were relatively dense and evenly distributed across the conterminous U.S. The NTN summary also included stations in Alaska and western Canada (Fig. 1). Of the 413 stations from both networks, 10 stations had no geographic coordinates or were located outside the study area. An additional 47 stations collected no data over the 1990–2013 study period, leading to a final sample size of 357 stations with 91,634 monthly observations. On average, stations were missing 42.1% of monthly measurements over 1990–2013. There were 122 stations missing fewer than 5% of monthly measurements.

### 1.2 Dry deposition

Estimates of dry N deposition were drawn from a study that integrated station measurements with satellite observations [3]. Maps of dry N deposition were received representing the sum of dry deposition of NO_2_, HNO_3_, $N{H_{4}}^{+}$, and $N{O_{3}}^{-}$. The method estimated ground N concentrations using NO_2_ satellite measurements from the Ozone Monitoring Instrument and ground measurements. As NH_3_ deposition flux could not be evaluated by NO_2_ columns, the map did not include deposition of NH_3_. The map was received at 0.25-degree spatial resolution and expressed the mean annual flux density for 2005–2014.

### 1.3 Pre-industrial total deposition

Estimates of total N deposition in 1860 were taken from a global model [4], [5]. The “Global Maps of Atmospheric Nitrogen Deposition, 1860, 1993, and 2050” database was downloaded from ORNL-DAAC (daac.ornl.gov/CLIMATE/guides/global_N_deposition_maps.html). Data were originally given in mg N m^−2^ yr^−1^ and converted to kg N ha^−1^ yr^−1^. The annual maps were originally produced at a resolution of 5.00° latitude and 3.75° longitude.

### 1.4 Precipitation

Long-term (1971–2000) monthly normal precipitation depths were derived from the ClimateNA software package [6]. Monthly deviations from the long-term monthly normal precipitation (i.e., anomalies) were developed from 1990 to 2013 based on station measurements from Environment Canada's online daily climate archive and from the U.S. Historical Climate Network [7].

## 2 Experimental design, materials, and methods

### 2.1 Processing

The database was developed at 1-km resolution in a Lambert conformal conic projection. Land regions outside of Alaska, the conterminous U.S. and Canada were not included. Monthly long-term normal precipitation depths were constructed by running the ClimateNA software package for the 1 km grid. Precipitation anomalies for each and year of the record were then produced from spatial interpolation of the station measurements. Final values of actual precipitation for each month (mm month^−1^) were then calculated by adding the normal for each month and the anomaly for each year and month.

**Fig. 2:**
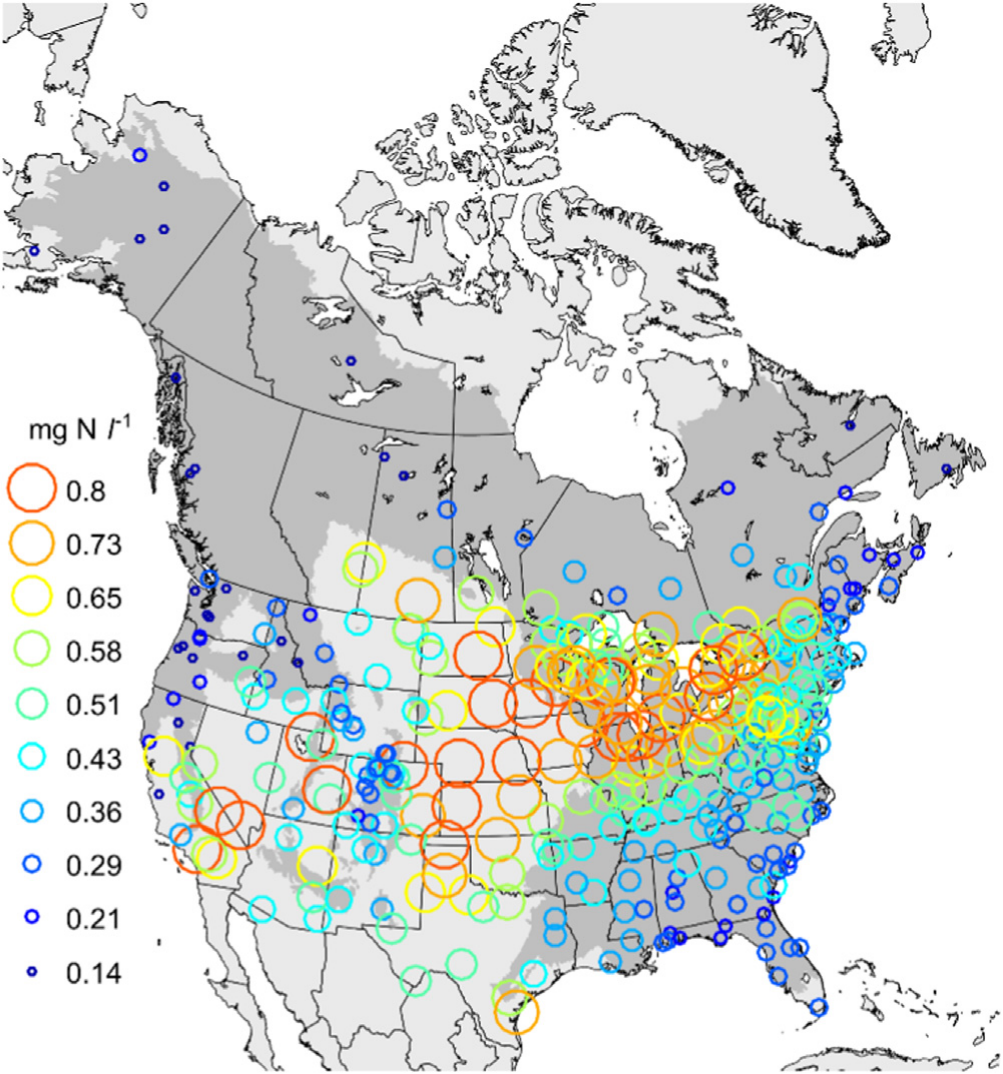
Mean concentrations of nitrogen in precipitation for available periods between 1990 and 2013. Values were binned by 10-percentile intervals between the 5th and 95th percentiles.

For each station record, monthly mean concentrations of $N{H_{4}}^{+}$ and $N{O_{3}}^{-}$ were standardized to units of mg N l^−1^ based on the molecular weight ratio of N in each compound and then combined to form total concentration of N in solution. Mean N concentrations for the available measurements at each station over 1990–2013 ranged from 0.10 to 0.90 mg N l^−1^ (Fig. 2). The point-estimates of N concentration for each combination of month and year were spatially interpolated to a 1 km grid covering North America. For each month, the interpolation step fit a polynomial surface to the measurement locations. Values of wet deposition in mg m^−2^ were then calculated by multiplying N concentrations by the corresponding grids of precipitation depth. Annual wet N deposition (kg N ha^−1^ yr^−1^) was then calculated by multiplying deposition rates by a 1×10^−6^ kg mg^−1^, multiplying by 1×10^4^ m^2^ ha^−1^, and summing monthly values for January–December for each year. The dry deposition from [3] was reprojected from geographic coordinates to that of the study coordinate system and then interpolated to the 1-km resolution grid using cubic polynomial fits.

To produce the best-possible approximation over the study period, 1990–2013, we calculated the mean annual wet N deposition using the values between 2005 and 2013 for comparison with the 2005–2014 mean dry deposition available from [3], accepting the error arising from the one-year discrepancy between averaging periods of each variable. We then calculated the mean dry:wet N deposition ratio (DWR) for this period. Total N deposition for each year, *t*, during 1990–2013 was then calculated as:

(1) $$N_{\mathrm{tot}}\left(t\right)=N_{\mathrm{wet}}\left(t\right)+\mathrm{DWR}\times \;N_{\mathrm{wet}}\left(t\right)$$

Hence, for the 1990–2013 period, spatial and temporal variation in wet N deposition is based on explicit annual measurements, while dry N deposition is based on the record-mean ratio, as informed by the mean spatial variation reported by [3]. Total N deposition for the year 1860 was calculated by reprojecting and interpolating model estimates across the study area at 1-km resolution using nearest-neighbour interpolation. To interpolate annual values of total N deposition between 1861 and 1989, we assumed that total N deposition scaled with the increase in global anthropogenic CO_2_ emissions for each year between 1861 and 1989.

### 2.2 Quality assurance measures

**Fig. 3:**
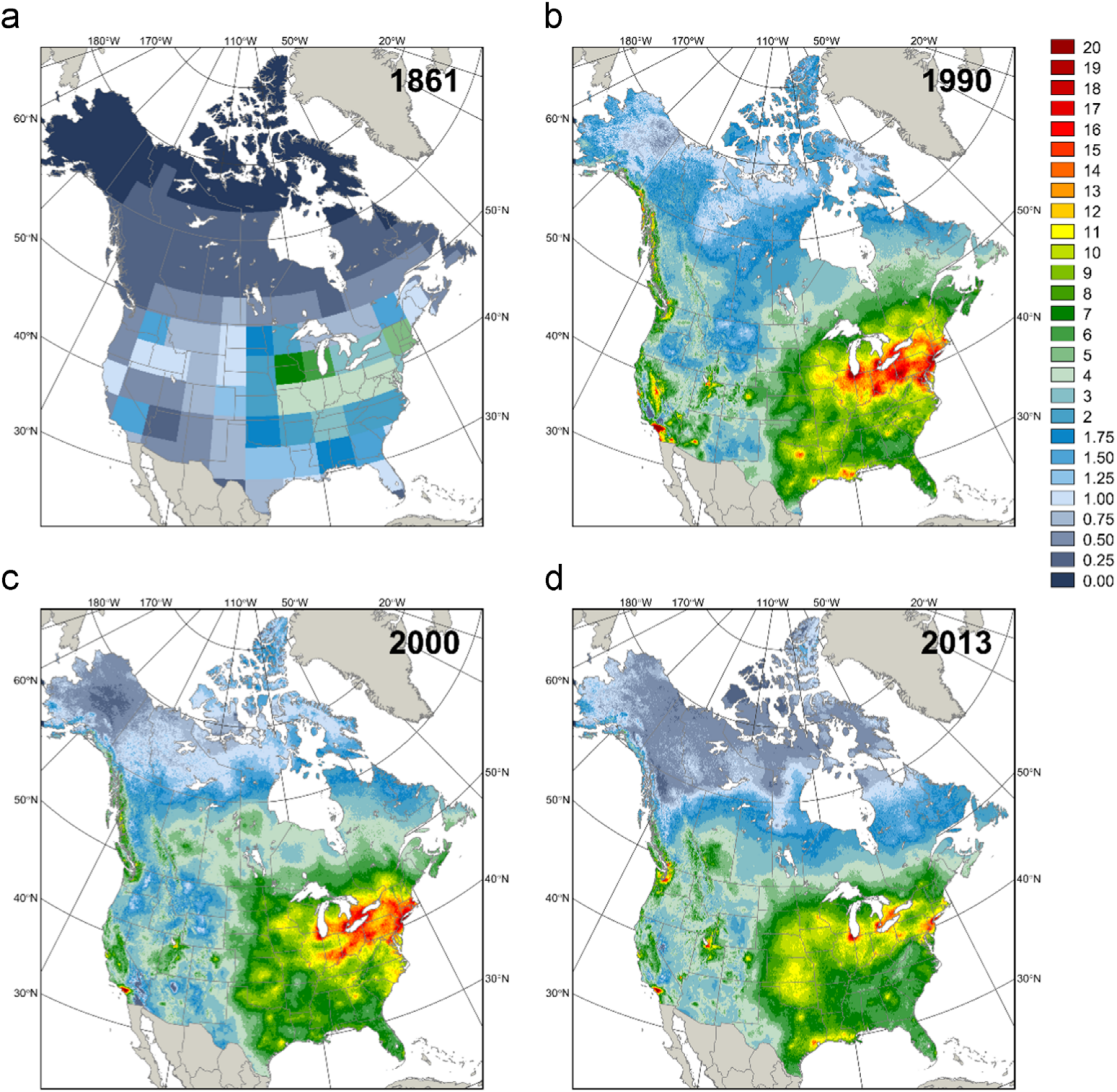
Estimates of annual total N deposition in (a) 1861 (b) 1990 (c) 2000 and (d) 2013 based on a combination of data sources, including the Canadian Air and Precipitation Monitoring Network (CAPMoN) measurements, National Atmospheric Deposition Program (NADP) measurements, estimates of dry N deposition from [3] and estimates of pre-industrial total N deposition from [4], [5].

As a basic measure to identify inconsistencies with the final integrated dataset, estimates of the long-term mean annual total N deposition were summarizing by North American ecozone [8] (Table 1) and maps of annual total N deposition were produced for the first year of the record and three years indicating the start, midpoint, and last year of the period with discontinuous ground measurements (Fig. 3).

**Table 1:** Descriptive statistics of annual total N deposition over 1990–2013 summarized by level 1 ecozones in Canada and the U.S. Standard deviations express variation across grid cells rather than across years. Trends were calculated from least-squares fits to time: (N_tot2013_ - N_tot1990_)/N_tot1990_ ×100, where N_tot1990_ and N_tot2013_ are predictions from the regression model.

	Total deposition (kg N ha^−1^ yr^−1^)		1990–2013 trend
	Mean	S.D.	(%)
Arctic Cordillera, CA	1.15	0.21	−36.1
Northern Arctic, CA	1.18	0.27	−41.1
Southern Arctic, CA	1.22	0.22	−22.4
Taiga Plain, CA	1.17	0.24	−18.2
Taiga Shield, CA	2.13	0.39	−27.1
Boreal Shield, CA	4.27	0.59	−21.0
Atlantic Maritime, CA	4.86	0.83	−38.0
Mixedwood Plain, CA	10.51	1.41	−28.0
Boreal Plain, CA	2.93	0.45	23.2
Prairie, CA	3.59	0.45	23.3
Taiga Cordillera, CA	1.16	0.34	−36.2
Boreal Cordillera, CA	1.42	0.42	−50.5
Pacific Maritime, CA	4.94	0.75	−30.4
Montane Cordillera, CA	2.66	0.35	−10.7
Hudson Plain, CA	3.57	0.67	−16.7
Marine West Coast Forest, US	4.96	0.82	−13.6
North American Deserts, US	3.11	0.47	−15.5
Northwestern Forested Mountains, US	3.59	0.44	−12.1
Temperate Sierras, US	3.91	0.85	9.9
Great Plains, US	6.18	0.41	7.5
Eastern Temperate Forests, US	9.22	0.90	−18.7
Northern Forests, US	7.79	1.01	−25.0
Mediterranean California, US	6.51	1.88	−28.7
Tropical Wet Forests, US	6.08	0.80	−11.5
Southern Semi-arid Highlands, US	3.47	0.87	25.1
